# Study on Data Preprocessing for Machine Learning Based on Semiconductor Manufacturing Processes

**DOI:** 10.3390/s24175461

**Published:** 2024-08-23

**Authors:** Ha-Je Park, Yun-Su Koo, Hee-Yeong Yang, Young-Shin Han, Choon-Sung Nam

**Affiliations:** 1 Department of Software Convergence Engineering, Inha University, 100 Inha-ro, Michuhol-gu, Incheon 22212, Republic of Korea; tomorrow0422@naver.com (H.-J.P.); gmldud6128@naver.com (H.-Y.Y.); 2 Department of Mechatronics Engineering, Inha University, 100 Inha-ro, Michuhol-gu, Incheon 22212, Republic of Korea; yskoo@inha.edu; 3 Frontier College, Inha University, 100 Inha-ro, Michuhol-gu, Incheon 22212, Republic of Korea

**Keywords:** SECOM dataset, semiconductor manufacturing process, machine learning, geometric mean, oversampling

## Abstract

Various data types generated in the semiconductor manufacturing process can be used to increase product yield and reduce manufacturing costs. On the other hand, the data generated during the process are collected from various sensors, resulting in diverse units and an imbalanced dataset with a bias towards the majority class. This study evaluated analysis and preprocessing methods for predicting good and defective products using machine learning to increase yield and reduce costs in semiconductor manufacturing processes. The SECOM dataset is used to achieve this, and preprocessing steps are performed, such as missing value handling, dimensionality reduction, resampling to address class imbalances, and scaling. Finally, six machine learning models were evaluated and compared using the geometric mean (GM) and other metrics to assess the combinations of preprocessing methods on imbalanced data. Unlike previous studies, this research proposes methods to reduce the number of features used in machine learning to shorten the training and prediction times. Furthermore, this study prevents data leakage during preprocessing by separating the training and test datasets before analysis and preprocessing. The results showed that applying oversampling methods, excluding KM SMOTE, achieves a more balanced class classification. The combination of SVM, ADASYN, and MaxAbs scaling showed the best performance with an accuracy and GM of 85.14% and 72.95%, respectively, outperforming all other combinations.

## 1. Introduction

The semiconductor industry is a high-value-added sector in modern society. Semiconductors are essential components for products ranging from small electronic devices, such as PCs, smartphones, and tablets to large electronic devices like cars [1,2].

The semiconductor manufacturing process involves producing silicon wafers, fabricating integrated circuits, packaging these integrated circuits, assembling the final products, and conducting tests [3]. In such a process, predicting good and defective products during manufacturing is essential to enhance productivity and reduce costs [3,4].

SECOM [5], provided by UCI (University of California, Irvine, CA, USA), consists of real data generated from semiconductor manufacturing processes and is used for research in classifying good and defective products. The dataset contains 41,951 missing values, accounting for approximately 4.54% of the total data. Excluding time and labels, it comprises 590 features in total. The dataset is relatively small, with only 1568 instances, including 1464 instances of the good product class and 104 instances of the defective product class. This results in a highly imbalanced dataset, with a class ratio of approximately 1:0.07 between good and defective products.

Classifying good and defective products using semiconductor manufacturing process data, such as SECOM, require removing and estimating missing values and reducing dimensionality [6]. In addition, the class ratio of training data should be balanced by resampling before machine learning [7]. Without such preprocessing steps, machine learning results in low data classification performance [8,9]. Finally, when evaluating the model performance, it is essential to use evaluation metrics such as the geometric mean (GM) of sensitivity and specificity in addition to the traditional accuracy metric. Accuracy means the results are biased towards the majority class [10,11].

The process described above, if carried out without split training and testing data before preprocessing and data analysis such as handling missing values and dimension reduction, would result in the leakage of information from the testing data into the training data, leading to compromised data integrity and overfitting of the test data [12].

In recent years, previous research has utilized SECOM for classifying good and defective products in semiconductor manufacturing processes. Three dimensionality reduction methods, including Boruta, Multivariate Adaptive Regression Splines, and principal component analysis (PCA), were compared and analyzed in combination with the undersampling and oversampling techniques in [4]. The performance of Correlation-Based Feature Selection, PCA, and cases without dimensionality reduction was compared in [13]. After reducing dimensionality using Recursive Feature Elimination, various models were compared and analyzed in [14]. These studies primarily focus on dimensionality reduction and resampling techniques using SECOM. However, the methods and timing of separating training and test data differ across studies, and the lack of confusion matrices makes it difficult to verify the possibility of biased results toward certain classes. They did not use or could not be calculated using the geometric mean. Therefore, it is difficult to directly compare and evaluate previous studies. Specifically, in [13], oversampling was applied to the entire dataset, leading to information from the training data leaking into the test data and resulting in the inclusion of non-existent data in the test set, which poses a significant issue. All three studies [4,13,14] used a Normalizer for scaling and did not investigate whether the application of different scaling methods could potentially improve model performance. We separate the training and test data, apply resampling only to the training data, and use the geometric mean to evaluate the classification performance of each class in a balanced manner. Finally, we investigated various scaling methods to identify the most suitable scaling technique for each model.

Thus, this study classified good and defective products during the semiconductor manufacturing process utilizing semiconductor manufacturing process data and machine learning. SECOM is utilized for this purpose. Furthermore, SECOM was split into training and testing data, and research on dataset integrity was conducted. This research examined methods for handling missing values and dimension reduction techniques to decrease the features used in machine learning for semiconductor manufacturing process data, such as SECOM. In addition, this study examined resampling methods and performance evaluation techniques for imbalanced data.

## 2. Related Research

### 2.1. Missing Value

Missing values occur in the real world for various reasons. For example, survey respondents may fail to answer specific questions, and participants in clinical trials may drop out due to drug allergies or death. Moreover, manual data entry can result in input errors [7]. Semiconductor manufacturing process data can also contain missing values for various reasons, such as machine errors, sensor malfunctions, data transmission errors, and mistakes by facility managers.

Data analysis results can be skewed if missing values are not handled, and certain patterns of missing data can introduce bias during machine learning, reducing the generalization performance of a model [8]. This issue can be addressed by removing samples or features with missing values or replacing missing values with specific values [7,8]. On the other hand, removing missing values can lead to the loss of important information, potentially affecting the learning process. Moreover, deleting samples might be more beneficial for the learning process than replacing them with specific values when the proportion of missing values is high. Nevertheless, imputing specific values in place of missing values, such as the mean, is used when the proportion of missing values for each feature is low. This approach allows the model to utilize the characteristics of the remaining data for learning.

### 2.2. Dimensionality Reduction

SECOM is a high-dimensional dataset with 590 features. In high-dimensional data, the number of features used in machine learning increases, leading to the curse of dimensionality. The curse of dimensionality refers to the phenomenon where, as the number of dimensions increases, the distance between data points increases, reducing density and increasing noise, decreasing the generalization performance of the model and causing overfitting. In addition, the computational burden on the model increases, leading to longer training times. Therefore, reducing the dimensionality of high-dimensional data to a lower-dimensional space is necessary to address the curse of dimensionality by reducing the number of features [15].

Methods for dimensionality reduction include feature selection and feature extraction [15]. Feature selection involves choosing features from existing ones based on an analysis of the correlation between independent and dependent variables. This method has lower computational complexity and better generalization characteristics but ignores the dependencies between features [16]. Principal component analysis (PCA) is a representative feature extraction method that generates new features by extracting the principal components to maintain maximum variance in the data. PCA preserves as much information as possible from the original features before dimensionality reduction, but it assumes that the data are embedded in a globally or approximately linear low-dimensional space [15].

### 2.3. Handling Data Imbalance

Imbalanced data refers to datasets where the class distribution is not uniform. SECOM is also an imbalanced dataset, with a higher proportion of good product classes than defective ones. Imbalanced datasets cause a lack of information in the minority class, leading to a bias towards the majority class, and poor classification performance in machine learning [6,9]. Therefore, resampling the data to balance the class proportions is necessary to mitigate this.

Resampling methods include undersampling, which reduces the majority class samples to match the minority class samples, and oversampling, which increases the minority class samples to match the majority class samples [10,17]. Both methods can yield better results than the original dataset. On the other hand, undersampling may result in a loss of essential sample data, while oversampling increases the dataset size, leading to higher computational costs [10].

### 2.4. Scaling

SECOM does not disclose which equipment was used for data collection. Similarly, semiconductor manufacturing process data are collected using various types of equipment, resulting in different units and ranges for the collected data. In machine learning, the results can be biased towards data with larger units or wider ranges if the maximum, minimum, or distribution of the data varies, degrading the performance [18].

Preventing such bias requires scaling the data to match the ranges. Scaling methods include normalization and standardization [19]. Standardization transforms the data such that the mean is 0 and there is a standard deviation of 1. Normalization adjusts the data range to be within 0 and 1, or −1 and 1, or other specified ranges.

### 2.5. Model Classification Performance Metrics

Model classification performance evaluation metrics include various methods such as accuracy and precision [10]. These metrics are calculated based on the confusion matrix and indicate the classification performance of the model. In binary classification problems, the confusion matrix consists of true negative (TN), false positive (FP), false negative (FN), and true positive (TP) as listed in Table 1. Each element provides information about the predictions and outcomes of the model. A TN indicates that the model predicted a negative result, and the actual result was also negative; hence, the result is true. An FP indicates that the model predicted positive, but the actual result was negative; hence, the result is false. An FN indicates that the model predicted negative, but the actual result was positive; hence, the result is false. A TP indicates that the model predicted positive, and the actual result was positive; hence, the result is true.

Accuracy represents the proportion of true outcomes in the overall predictions of the model, reflecting the classification performance across all classes, as expressed in Equation (1) [10,11]. In a dataset with imbalanced classes, if all predictions for the negative class are true and for the positive class there are five true and five false predictions, the accuracy still appears as 99%, indicating a bias towards the majority class, as shown in the confusion matrix in Table 2. In this scenario, the accuracy for the positive class was only 50%, but the overall accuracy was 99%. Therefore, for datasets with uneven class distributions, metrics, such as sensitivity and specificity, should be used alongside accuracy to evaluate the classification performance of the model [10].

(1) $$Accuracy=\frac{\left(TN+TP\right)}{\left(TN+FP+FN+TP\right)}$$

Sensitivity (also known as recall) is the proportion of true outcomes for the positive class predictions (Equation (2)), and specificity is the proportion of true outcomes for the negative class predictions, as shown in Equation (3) [10]. The sensitivity and specificity reflect the classification performance of the model for a single class. On the other hand, using the results for only one class does not provide a complete picture of the performance of a model across all classes. Therefore, the GM of the sensitivity and specificity should be used to address this issue [10,11].

(2) $$Sensitivity=\frac{TP}{\left(TP+FN\right)}$$

(3) $$Specificity=\frac{TN}{\left(TN+FP\right)}$$

Equation (4) expresses the GM, which is determined by using sensitivity and specificity to include the prediction results for two classes and then taking the square root to calculate the geometric mean of the ratio between sensitivity and specificity. For example, when sensitivity and specificity are 50% and 100%, respectively, as shown in Table 2, the GM is 70.7%, reflecting the balanced performance of the model with imbalanced data. Therefore, when evaluating the classification performance of models using imbalanced datasets, such as semiconductor manufacturing process data, it is important to use metrics, such as GM, in addition to accuracy to assess classification performance of the model.

(4) $$Geometric\;Mean=\sqrt{Sensitivity\times Specificity}$$

## 3. Research Method

This paper examined the optimal combination of preprocessing methods and machine learning models using SECOM to predict good and defective products from semiconductor manufacturing process data. In addition, it conducted a comparative evaluation against the project by Samsung SDS [20].

**Figure 1:**
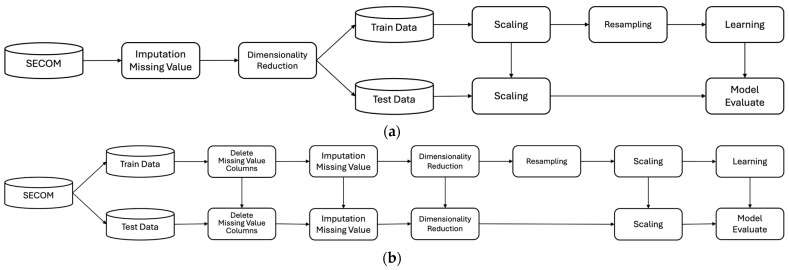
Research processes: (**a**) Samsung; (**b**) this paper.

Figure 1 shows the research processes of Samsung and this paper. As shown in Figure 1a, Samsung performed preprocessing, such as replacing missing values and dimensionality reduction, without separating the training and test data. However, unlike Samsung and previous studies, this paper assumed that if the separation of training and test data is not conducted as the first step, information from the training data could leak into the test data during data analysis and preprocessing, leading to overfitting of the model. Thus, this paper separated the data into training and test data at a ratio of 7 to 3 to ensure data integrity, as shown in Figure 1b and detailed in Table 3. Missing values were removed or replaced to clean the data, and the number of features was reduced for dimensionality reduction. The model was evaluated after training by resampling the training data to uniformly adjust the class ratio before applying machine learning. This study used the following six models: Logistic Regression (LR), Support Vector Machine (SVM), K Nearest Neighbor (KNN), Decision Tree (DT), Random Forest (RF), and Extreme Gradient Boosting (XGB). These models demonstrated improved performance through preprocessing of imbalanced datasets, such as those from semiconductor manufacturing processes [21,22,23].

### 3.1. Data Cleaning and Dimensionality Reduction

**Figure 2:**
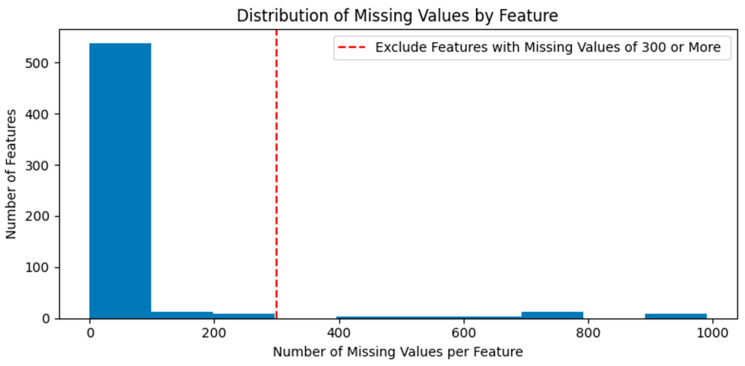
Distribution of missing values by feature.

Before processing the raw data, SECOM was divided into training and test data. Missing values were deleted or replaced based on the training data. Figure 2 presents the distribution of the number of missing values according to the feature in the training data. After deleting 31 features with more than 300 missing values per feature, the remaining missing values were replaced with feature-specific averages. The test data also had the same features deleted as in the training data, and missing values were replaced with the feature-specific averages from the training data. This study assumed that the independent and dependent variables have a linear relationship because SECOM does not provide the type, form, and unit of collected data. Therefore, missing values were replaced with feature-specific averages to reduce their effect on machine learning.

**Figure 3:**
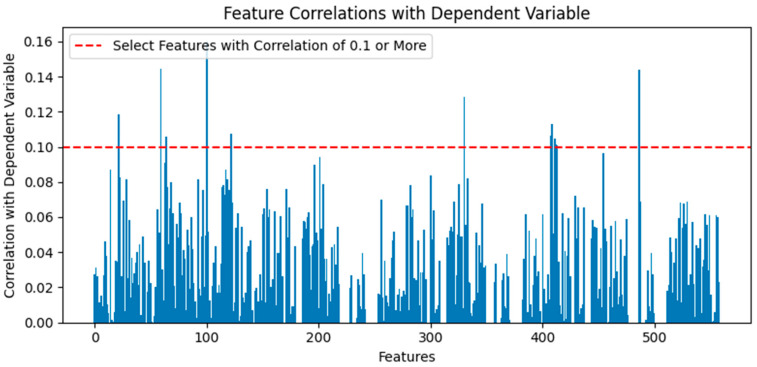
Feature correlations with dependent variable.

The dataset remained high-dimensional with 558 features, even after data cleaning. Feature selection methods using Pearson correlation coefficients were applied to reduce the number of features. A correlation coefficient above 80% indicates a very strong correlation, above 60% indicates a strong correlation, above 40% indicates a moderate correlation, and above 20% indicates a weak correlation [24]. In the case of SECOM, which contains 1568 total data instances, the number of instances per class is insufficient, resulting in very low correlation coefficients with the dependent variable. Since there were no features with a correlation coefficient above 20%, only those with a correlation coefficient above 10% were selected. The Pearson correlation coefficients between the independent and dependent variables were calculated based on the learning data. Absolute values were used to select sensors 21, 59, 64, 100, 122, 330, 407, 408, 411, 412, 413, and 486 with a correlation coefficient of 10% or more, reducing the dimension to 12 features and selecting the same features for the test data. Figure 3 shows the correlation coefficients by feature.

The machine learning model was trained after data cleaning and dimensionality reduction to compare the training data before and after resampling. The average accuracy of the five models, excluding DT, was 93.2%, with sensitivity biased towards 0% for good products, as listed in Table 4. DT has a lower accuracy of 88.11% compared to other models, but it shows a GM of 30.1%, which is higher than other models. However, the sensitivity remains low at 9.68%, indicating poor performance in classifying defective products. 

### 3.2. Resampling

The class imbalance problem in SECOM was addressed by applying effective oversampling methods, as demonstrated by the approach in [10]. The methods utilized included the Synthetic Minority Oversampling Technique (SMOTE), Adaptive Synthetic Sampling (ADASYN), Borderline SMOTE (BL SMOTE), K-Means SMOTE (KM SMOTE), and Support Vector Machine SMOTE (SVM SMOTE), all of which demonstrated improved performance after resampling [11,17,21]. Table 5 lists the ratio of good and defective products in the training data after applying each oversampling method.

Table 6 presents the test results after machine learning after oversampling the training data with an oversampling method and model. The combination of SVM, SMOTE, ADASYN, and BL SMOTE achieved the highest sensitivity at 70.97% based on sensitivity, as shown in Table 6. On the other hand, the average accuracy was 32.76%, indicating that the classification performance was biased towards defective products. The combination of LR and SMOTE classified good and bad products in a balanced manner, with a GM of 69.5%, the highest among all combinations. However, when using KM SMOTE with the RF and XGB models, the sensitivity does not increase even after oversampling. In the LR, KNN, and DT models, the sensitivity is significantly lower with KM SMOTE than with other oversampling methods: 19.35% for LR, 3.23% for KNN, and 12.9% for DT. However, in the case of SVM with SVM SMOTE, it has the lowest sensitivity at 6.45%. Therefore, since KM SMOTE results in biased outcomes toward good products compared to other oversampling methods, it is excluded from subsequent research. SVM SMOTE, on the other hand, is not excluded because it increased sensitivity in models other than SVM.

### 3.3. Scaling

**Figure 4:**
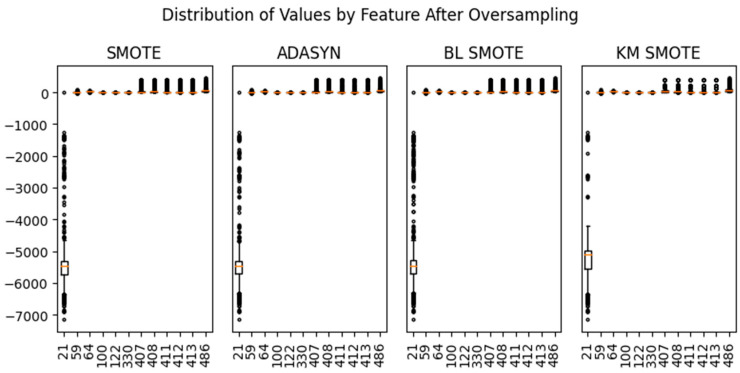
Distribution of values by feature after oversampling.

Figure 4 shows the value distribution by feature after oversampling. The range of the data is larger than that of other features in the case of sensor 21. Scaling was performed to match the range of the data using the Standard, MinMax, MaxAbs, Robust, and Normalizer [18].

**Figure 5:**
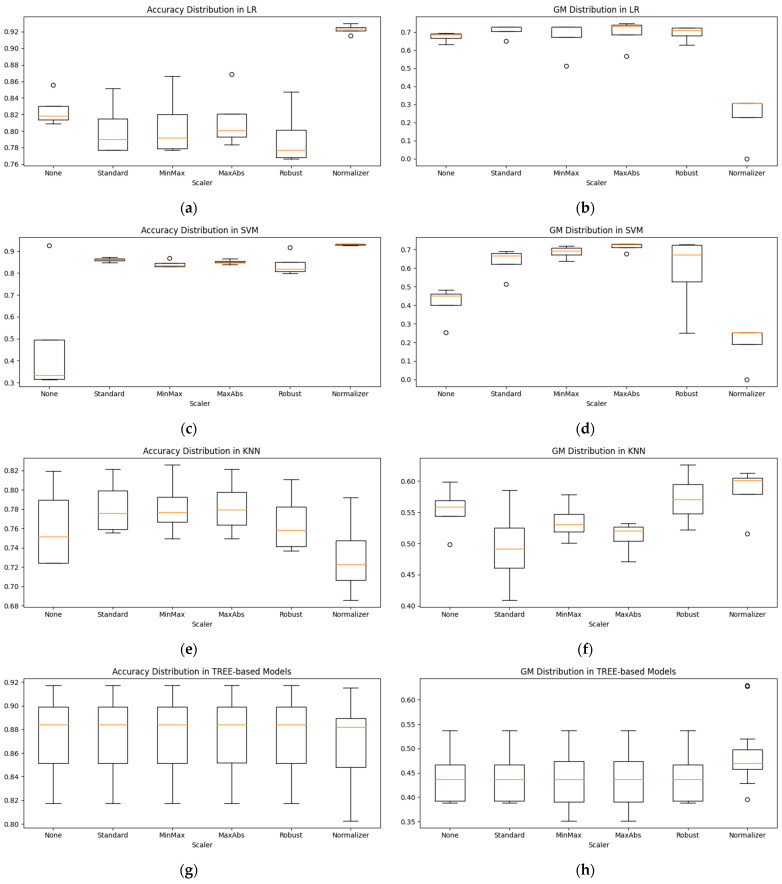
Accuracy and GM distribution before and after scaling by model: (**a**) accuracy distribution in LR; (**b**) GM distribution in LR; (**c**) accuracy distribution in SVM; (**d**) GM distribution in SVM; (**e**) accuracy distribution in KNN; (**f**) GM distribution in KNN; (**g**) accuracy distribution in Tree-based models; (**h**) GM distribution in Tree-based models.

Figure 5 shows the distribution of accuracy and the GM before and after scaling for each model after oversampling. Figure 5a,c show the distribution of accuracy after scaling for LR and SVM, and Figure 5b,d show the distribution of the GM after scaling of LR and SVM. The accuracy distribution increased compared to other scaling methods when LR and SVM used a Normalizer. On the other hand, in the case of the GM, the distribution decreased compared to that before the application of the Normalizer. For LR, the accuracy distribution after scaling decreased except for when using the Normalizer, but the GM distribution increased. MaxAbs achieved the highest GM and had a high median value. After scaling, SVM showed an increased distribution in the accuracy and GM, with MaxAbs being stable and achieving the highest GM because of the small distribution of accuracy and the GM. In the case of KNN, the accuracy distribution increased for scaling methods other than the Normalizer, as shown in Figure 5e. Figure 5f shows the distribution of the GM after scaling. The distribution of the Standard method is lower than before scaling, while the Normalizer has the highest median value and is stable because of its narrow distribution, although the highest value is achieved with Robust scaling. Tree-based models, such as DT, RF, and XGB, show a slight decrease in accuracy with the Normalizer, as shown in Figure 5g, but other scaling methods result in a slight change compared to before scaling. The Normalizer increased the distribution and maximum value of the GM as shown in Figure 5h. Thus, the scaling results vary from model to model; a single scaling method cannot be applied universally to all models. Therefore, this study selected a combination of the model, oversampling method, and scaling method by applying all scaling methods.

### 3.4. Comparative Evaluation

The results of this research were compared with the project process and results of Samsung. The biggest difference is that in the case of Samsung, missing values processing and dimensionality reduction were performed before the training data and test data were separated. In addition, Samsung selected features with a correlation coefficient of 6.9% or more and reduced them to 40 features. In this study, missing value processing and dimensionality reduction were carried out after separating the training data and the test data. In addition, the dimension was reduced to 12 features with a correlation coefficient of 10% or more.

**Figure 6:**
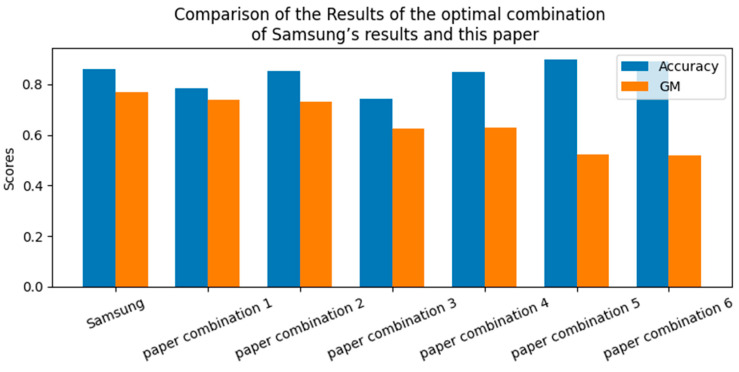
Comparison of the results of the optimal combination of Samsung’s results and this paper.

Table 7 and Figure 6 list the results of Samsung and the optimal combination results of this paper by model. In the case of Samsung, the combination of XGB, SMOTE, and Normalize showed 85.99% accuracy and 76.9% GM. The best combination in this study was SVM, ADASYN, and MaxAbs, with an accuracy of 85.14%, which was 0.85% lower than that of Samsung, and a GM of 3.95%. On the other hand, the training time and prediction time of the model were reduced via the machine learning process using 12 features, 28 fewer features than Samsung. In addition, the process of this study suggests that the model was less likely to overfit the test data, and the generalization performance for the new test data was higher than Samsung’s process because the training data and the test data were separated and then missing value processing and dimensionality reduction were performed.

## 4. Conclusions

The semiconductor industry is one of the most highly valued industries for the future in modern society, making it crucial to accurately classify good and defective products in the semiconductor manufacturing process. The semiconductor manufacturing process is highly complex, continuously generating hundreds of types of measurement data to improve yield. Using this vast amount of measured semiconductor manufacturing process data is essential to automated quality classification because it can significantly enhance the efficiency of the manufacturing process.

The purpose of this study is to improve the efficiency of defect detection by predicting defective products in advance using data generated from the semiconductor manufacturing process. To achieve this, this paper investigates methods for predicting defective products during the semiconductor manufacturing process using machine learning. This paper analyzed semiconductor manufacturing process data based on SECOM, focusing on handling missing values, dimensionality reduction, and resampling preprocessing techniques, with the aim of improving the efficiency of the semiconductor manufacturing process. 

We compared and analyzed the results of 150 different combinations by utilizing six models, five oversampling methods, and five scaling methods for this study. As a result, the model that utilized SVM with ADASYN for oversampling and MaxAbs for scaling demonstrated the highest performance, with an accuracy of 85.14% and GM of 72.95%. This result highlights the importance of the combination of resampling methods, scaling techniques, and models in dealing with imbalanced data.

In the case of Samsung, the XGB model, when scaled using Normalizer and oversampled with SMOTE, achieved an accuracy of 85.99% and GM of 76.9%, which is slightly higher than the results of this study. However, since the process of Samsung involved handling missing values and performing dimensionality reduction before separating the training and test data, this may have influenced the learning process and the resulting outcomes. In contrast, we separated the training and test data before handling missing values and performing dimensionality reduction, ensuring that the test data remained unaffected. The proposed method allows for a more objective evaluation and demonstrates that the classification performance of the model, as measured by the GM, was not biased toward either the good or defective class. Our proposed method achieved similar accuracy using only 12 features, which is less than Samsung’s 40 features. In other words, it was confirmed that this method is capable of efficiently predicting defective products with minimal features. From the results of analyzing various combinations of scaling methods and models, we confirmed that one scaling method does not consistently improve the performance of all models. We analyzed the optimal scaling methods that contribute to performance improvement for each model, and proposed scaling techniques that can maximize performance specific to each model. 

The machine learning models trained on our preprocessed data did not achieve an accuracy of over 90%. Thus, it is expected that future research could attain higher accuracy using KNN for missing data imputation, machine learning-based dimensionality reduction techniques, deep learning methods, and hyperparameter optimization. Finally, it is necessary to verify the generalizability of this research method using other semiconductor manufacturing process data.

## Figures and Tables

**Table 1:** Confusion matrix for binary classification.

		Predict	
		Negative	Positive
**Actual**	**Negative**	TN	FP
	**Positive**	FN	TP

**Table 2:** Confusion matrix for imbalanced data.

		Predict	
		Negative	Positive
**Actual**	**Negative**	1000	0
	**Positive**	5	5

**Table 3:** Class ratio of training data to test data.

	Training Data	Test Data
Good	1023	440
Defective	73	31
Ratio	1:0.07	

**Table 4:** Model-specific results after data cleaning and dimensionality reduction.

Model	Accuracy	GM	Sensitivity	Specificity
LR	0.9299	0	0	0.9955
SVC	0.9342	0	0	1
KNN	0.9342	0	0	1
DT	0.8811	0.3010	0.0968	0.9364
RF	0.9342	0	0	1
XGB	0.9278	0	0	0.9932

**Table 5:** Number and ratio of good and defective products after oversampling.

	SMOTE	ADASYN	BL SMOTE	KM SMOTE	SVM SMOTE
Good	1023	1023	1023	1023	1023
Defective	1023	999	1023	1024	595
Ratio	1:1	1:0.98	1:1	1:1	1:0.58

**Table 6:** Application of the oversampling methods by model.

Model	Sampling	Accuracy	GM	Sensitivity	Specificity
LR	SMOTE	0.8153	0.6950	0.5806	0.8318
	ADASYN	0.8089	0.6921	0.5806	0.8250
	BL SMOTE	0.8217	0.6791	0.5484	0.8409
	KM SMOTE	0.8429	0.4147	0.1935 *	0.8886
	SVM SMOTE	0.8556	0.6319	0.4516	0.8841
SVM	SMOTE	0.3163	0.4526	0.7097	0.2886
	ADASYN	0.3142	0.4508	0.7097	0.2864
	BL SMOTE	0.3524	0.4819	0.7097	0.3273
	KM SMOTE	0.8386	0.3781	0.1613	0.8864
	SVM SMOTE	0.9257	0.2523	0.0645 *	0.9864
KNN	SMOTE	0.7240	0.5988	0.4839	0.7409
	ADASYN	0.7240	0.5591	0.4194	0.7455
	BL SMOTE	0.7792	0.5589	0.3871	0.8068
	KM SMOTE	0.9299	0.1790	0.0323 *	0.9932
	SVM SMOTE	0.8195	0.4988	0.2903	0.8568
DT	SMOTE	0.8174	0.5243	0.3226	0.8523
	ADASYN	0.8301	0.4439	0.2258	0.8727
	BL SMOTE	0.8556	0.5368	0.3226	0.8932
	KM SMOTE	0.8832	0.3476	0.1290 *	0.9364
	SVM SMOTE	0.8386	0.4462	0.2258	0.8818
RF	SMOTE	0.8960	0.4928	0.2581	0.9409
	ADASYN	0.9023	0.4293	0.1935	0.9523
	BL SMOTE	0.9045	0.3928	0.1613	0.9568
	KM SMOTE	0.9324	0.0000	0.0000 *	1.0000
	SVM SMOTE	0.9172	0.3956	0.1613	0.9705
XGB	SMOTE	0.8832	0.3881	0.1613	0.9341
	ADASYN	0.8832	0.4581	0.2258	0.9295
	BL SMOTE	0.8854	0.3886	0.1613	0.9364
	KM SMOTE	0.9172	0.0000	0.0000 *	0.9818
	SVM SMOTE	0.8981	0.3914	0.1613	0.9500

* Worst sensitivity by sampling method.

**Table 7:** Results of the optimal combination of Samsung’s results and this paper by model.

	Model	Sampling	Scaler	Accuracy	GM
Samsung	XGB	SMOTE	Normalize	0.8599	0.7690
paper combination 1	LR	SMOTE	MaxAbs	0.7834	0.7383
paper combination 2 *	SVM	ADASYN	MaxAbs	0.8514	0.7295
paper combination 3	KNN	SMOTE	Robust	0.7431	0.6259
paper combination 4	DT	BL SMOTE	Normalize	0.8493	0.6294
paper combination 5	RF	SMOTE	MaxAbs	0.8981	0.5227
paper combination 6	XGB	BL SMOTE	Normalize	0.8896	0.5201

* Best combination in the paper.

## Data Availability

Data are contained within the article.
